# VECTOR: An Integrated Correlation Network Database for the Identification of CeRNA Axes in Uveal Melanoma

**DOI:** 10.3390/genes12071004

**Published:** 2021-06-29

**Authors:** Cristina Barbagallo, Antonio Di Maria, Adriana Alecci, Davide Barbagallo, Salvatore Alaimo, Lorenzo Colarossi, Alfredo Ferro, Cinzia Di Pietro, Michele Purrello, Alfredo Pulvirenti, Marco Ragusa

**Affiliations:** 1 Department of Biomedical and Biotechnological Sciences—Section of Biology and Genetics, University of Catania, 95123 Catania, Italy; barbagallocristina@unict.it (C.B.); adriana.alecci@hotmail.it (A.A.); dbarbaga@unict.it (D.B.); dipietro@unict.it (C.D.P.); purrello@unict.it (M.P.); 2 Department of Clinical and Experimental Medicine, University of Catania, c/o Dipartimento di Matematica e Informatica, Viale A. Doria 6, 95125 Catania, Italy; antoniodm@unict.it (A.D.M.); salvatore.alaimo@unict.it (S.A.); alfredo.ferro@unict.it (A.F.); mragusa@unict.it (M.R.); 3 Department of Experimental Oncology, Mediterranean Institute of Oncology (IOM), 95029 Catania, Italy; lorenzo.colarossi@grupposamed.com

**Keywords:** ncRNA, ceRNA, miRNA, lncRNA, bioinformatics, cancer, network

## Abstract

Uveal melanoma (UM) is the most common primary intraocular malignant tumor in adults and, although its genetic background has been extensively studied, little is known about the contribution of non-coding RNAs (ncRNAs) to its pathogenesis. Indeed, its competitive endogenous RNA (ceRNA) regulatory network comprising microRNAs (miRNAs), long non-coding RNAs (lncRNAs) and mRNAs has been insufficiently explored. Thanks to UM findings from The Cancer Genome Atlas (TCGA), it is now possible to statistically elaborate these data to identify the expression relationships among RNAs and correlative interaction data. In the present work, we propose the VECTOR (uVeal mElanoma Correlation NeTwORk) database, an interactive tool that identifies and visualizes the relationships among RNA molecules, based on the ceRNA model. The VECTOR database contains: (i) the TCGA-derived expression correlation values of miRNA-mRNA, miRNA-lncRNA and lncRNA-mRNA pairs combined with predicted or validated RNA-RNA interactions; (ii) data of sense-antisense sequence overlapping; (iii) correlation values of Transcription Factor (TF)-miRNA, TF-lncRNA, and TF-mRNA pairs associated with ChiPseq data; (iv) expression data of miRNAs, lncRNAs and mRNAs both in UM and physiological tissues. The VECTOR web interface can be queried, by inputting the gene name, to retrieve all the information about RNA signaling and visualize this as a graph. Finally, VECTOR provides a very detailed picture of ceRNA networks in UM and could be a very useful tool for researchers studying RNA signaling in UM. The web version of Vector is freely available at the URL reported at the end of the Introduction.

## 1. Introduction

Uveal melanoma (UM) is the second most common type of human melanoma and the most frequent primary tumor of the eye in adults, with an annual incidence of 6–7 cases per million per year [1]. It mainly affects the choroid and its early metastasis, mostly to the liver, leads to 50% of the death rate in patients [2]. Several molecular alterations have been associated with the development of UM; however, its etiology remains unclear. Monosomy of chromosome 3 and gain of 8q are often found in UM patients [3]. Similarly, UM progression is frequently linked to oncogenic mutations of some genes, such as G protein subunit α q (GNAQ), G protein subunit α 11 (GNA11) and BRCA associated protein 1 (BAP1), related to transcriptional, post-transcriptional and post-translational dysregulations impairing cell cycle and apoptosis [4]. Understanding UM tumorigenesis solely by investigating genetic mechanisms is too limiting. Epigenetic alterations may be considered an important hallmark of cancer because of their critical role in the initiation of tumorigenesis [5]; however, these mechanisms are not well characterized in the onset and progression of UM. Cancer-related epigenetic phenomena include altered methylation of oncogenes and tumor suppressor genes, irregular histone modification pattern and aberrant expression of non-coding RNAs (ncRNAs) [6,7]. This last issue has been extensively explored in the last two decades in the most common types of cancer. NcRNAs mainly include microRNAs (miRNAs) and long non-coding RNAs (lncRNAs). More specifically, miRNAs are 18–25 nucleotide RNA molecules inducing mRNA degradation or translation inhibition through binding to the miRNA response element (MRE) at 3′ untranslated regions (3′-UTRs) of mRNAs. LncRNAs are the most functionally heterogeneous class of ncRNAs, with lengths ranging from 200 nt to 100,000 nt. They also contain MREs and, accordingly, can sequester specific miRNAs preventing them from binding mRNAs. On the other hand, miRNA binding to lncRNAs could promote their decay, under specific stoichiometric conditions [8,9]. In other words, some lncRNAs and mRNAs can share the same MRE and compete for binding to the same miRNAs, creating, in this way, a competitive endogenous RNA (ceRNA) regulatory network. Through such a competitive mechanism of RNA-RNA interactions, increased levels of lncRNAs decrease the quantity of available miRNAs and prevent mRNA degradation or translation block. Conversely, downregulation of a specific lncRNA induces miRNA release and mRNA degradation or reduced translation [9]. This “miRNA sponge” function operated by lncRNAs has been described in many cancer models [10,11,12,13], including UM [14,15]. It has been reported that perturbations of the ceRNA network influence all the cellular signaling underlying cancer phenotypes; however, this topic is currently still poorly investigated in UM. With the advances in RNA sequencing technology and the emergence of public cancer genomics projects, such as The Cancer Genome Atlas (TCGA), a huge amount of transcriptomic data from tumor specimens has been generated and made publicly available for anyone in the research community to use. These data can be computationally elaborated and combined with other data of a different nature, such as RNA-RNA binding and regulation by transcription factors, to build ncRNA networks allowing researchers to in silico explore cancer-related RNA signaling. For this purpose, we calculated the expression correlation of miRNAs, lncRNAs and mRNAs from the UM TCGA dataset [16], consisting of 80 UM patients. This information was combined with data (where available) of RNA-RNA interactions, regulation by transcription factors, and genomic positions. All these data have been collected and stored in a novel database called VECTOR (uVeal mElanoma Correlation neTwORk). VECTOR, through specific RNA-species queries, can identify new RNA circuits and their regulation signaling in UM. VECTOR makes UM RNA networks available to cancer researchers who intend to computationally explore the potential cross-talking between ncRNAs and mRNAs before performing in vitro or in vivo experiments on ocular melanoma models.

## 2. Materials and Methods

### 2.1. Data Collection

All the normalized expression data about TCGA UM (80 tumor tissue samples) were extracted from the UCSC Xena Server (https://xena.ucsc.edu, accessed on 28 December 2020). Normalization was performed by the TCGA Consortium. MiRNA expression data of physiological tissues were retrieved from Human miRNA tissue atlas (https://ccb-web.cs.uni-saarland.de/tissueatlas/, accessed on 28 December 2020) [17], while mRNAs and lncRNAs expression data were from Expression Atlas (https://www.ebi.ac.uk/gxa/home, accessed on 28 December 2020) (accession E-MTAB-2836) [18,19,20]. Validated or predicted interactions between miRNAs and mRNAs were retrieved by DIANA-TarBase v8 (https://carolina.imis.athena-innovation.gr/diana_tools/web/index.php?r=tarbasev8%2Findex, accessed on 28 December 2020) [21] and miRTarBase (http://miRTarBase.cuhk.edu.cn, accessed on 28 December 2020) [22]; while, lncRNA:miRNA interaction data were from miRcode (www.mircode.org, accessed on 28 December 2020), Encori (http://starbase.sysu.edu.cn, accessed on 28 December 2020) [23], DIANA-LncBase v3 (https://diana.e-ce.uth.gr/lncbasev3, accessed on 28 December 2020) [24]. Information on binding of transcription factors to promoters of miRNAs, lncRNAs, mRNAs were extracted as ChiPseq data elaborated by TransmiR v2.0 (http://www.cuilab.cn/transmir, accessed on 28 December 2020) [25], ENCODE (https://www.encodeproject.org, accessed on 28 December 2020) [26] and ChEA (http://amp.pharm.mssm.edu/Enrichr, accessed on 28 December 2020) [27,28]. Genomic positions were retrieved by using the UCSC genome browser (https://genome.ucsc.edu, accessed on 28 December 2020) [29].

### 2.2. Data Elaboration

Expression data retrieved from the TCGA dataset were filtered. To avoid any statistical confounding effect, null expression values were excluded: we arbitrarily chose to maintain for successive analyses only RNAs showing expression values greater than 0 in at least 60 out of 80 samples (75% of samples). Then, expression correlation matrices based on the Pearson calculation were computed between (a) miRNAs and mRNAs, (b) miRNAs and lncRNAs, and (c) lncRNAs and mRNAs. Based on the “miRNA sponge model”, we considered those RNA axes characterized by the following mathematical correlation as consistent: miRNAx:lncRNAy (negative Pearson) + miRNAx:mRNAz (negative Pearson) + lncRNAy:mRNAz (positive Pearson). Separately, we extracted from the mRNA dataset the expression of transcription factors (TFs) according to the list deposited on The Human Transcription Factors website (humantfs.ccbr.utoronto.ca). We then computed the expression correlation matrix between TFs and all the other RNA classes, for each correlation coefficient, a *p*-value was calculated.

### 2.3. VECTOR Data and Architecture

**Figure 1:**
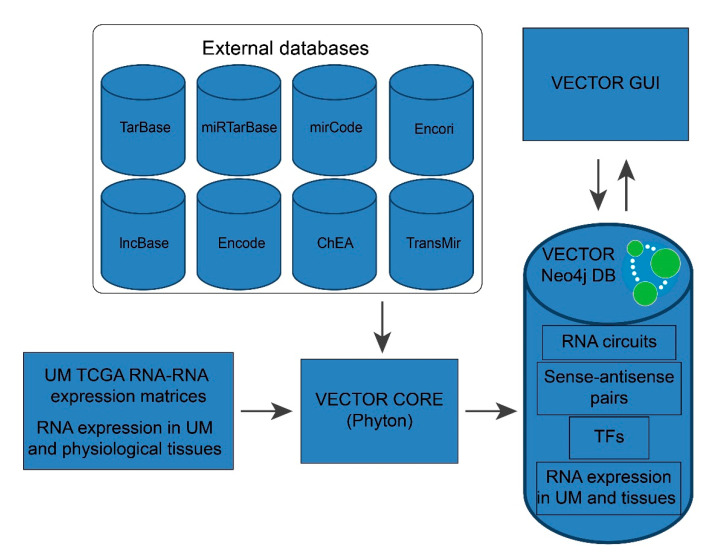
Architecture of VECT model. Cylinders represent databases (both external databases and VECTOR); rectangles depict graphical interface modules or database processing applications. Neo4j database logo is shown within the VECTOR database.

The VECTOR database has been built on top of the Laravel model-view-controller framework. All the data have been collected into a Neo4j database. Data processing has been carried out in R, Python and PHP. All the components of the Web Interface have been implemented in React native to ensure high modularity and dynamicity (Figure 1).

VECTOR stores the following information:

- The correlation values (Pearson coefficients) of miRNA-mRNA, miRNA-lncRNA and lncRNA-mRNA pairs in UM samples. These correlations are used to create correlation networks, which show feedback loops involving the three classes of RNAs. The above-mentioned pairs of molecules are associated with data coming from miRBase, miRTarBase, LncBase, miRcode and Encori databases, also storing information about the predicted or validated RNA-RNA interactions. All correlation values can be downloaded by users at the “Download” section.
- Overlapping of genomic positions between mRNAs and lncRNAs, in order to find couples of sense-antisense transcripts.
- Correlation coefficients of TF-miRNA, TF-lncRNA, and TF-mRNA pairs in UM samples. These TF:RNA couples were associated with ChiPseq data of TF binding from TransmiR, ENCODE, and ChEA, in order to corroborate the potential TF regulation on miRNAs, lncRNAs and mRNAs.
- The expression values of miRNAs, expressed as log2(RPM + 1), mRNAs and lncRNAs, expressed as log2(x + 1) normalized count, were retrieved from the TCGA dataset. Assitionally, VECTOR includes expression data of miRNAs, mRNAs and lncRNAs in several physiological tissues, reported as quantile normalized expression (miRNAs) and TPM (Transcripts Per Kilobase Million) (mRNAs and lncRNAs). Clinicopathological parameters of UM patients included in the UM TCGA dataset were collected and stored in VECTOR.

### 2.4. VECTOR Web Interface

**Figure 2:**
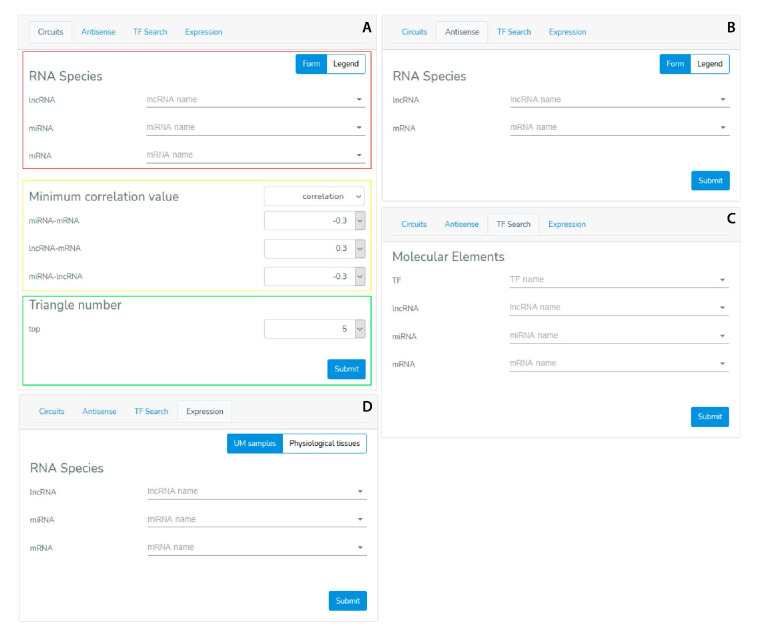
Menu section of the VECTOR database. (**A**) The “Circuit” menu queries the database by choosing at least an RNA molecule (lncRNA and/or miRNA and/or mRNA), a minimum correlation value for each pair and the number of network motifs to be shown. Alternatively, user can filter the output by *p*-value. (**B**) The “Antisense” menu identifies sense-antisense pairs by choosing a lncRNA and/or mRNA. (**C**) The “TF Search” menu retrieves information on potential TFs regulating lncRNA, miRNA or mRNA expression in UM by choosing a TF and/or a lncRNA, a miRNA, or a mRNA. (**D**) The “Expression” menu allows to inspect the expression of a chosen lncRNA, miRNA or mRNA in UM samples or several physiological tissues.

**Figure 3:**
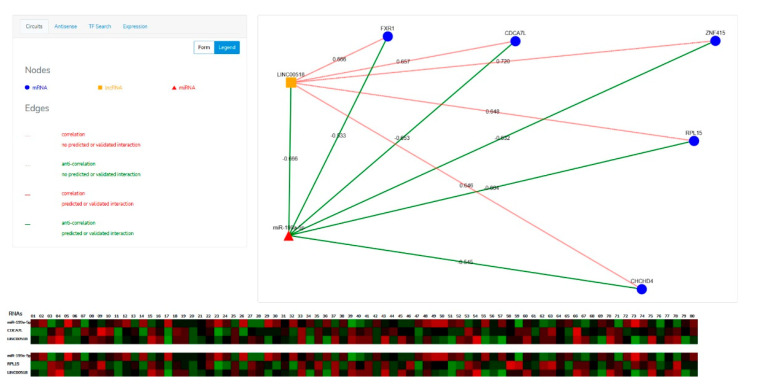
Result section of the VECTOR database for the “Circuits” query. Output data based on miRNA sponge activity of lncRNAs are depicted as an interactive network. UM expression data of each member of the circuits is shown as heatmap.

The GUI consists of two sections: the Menu section (Figure 2) and the Results section (Figure 3).

**Figure 4:**
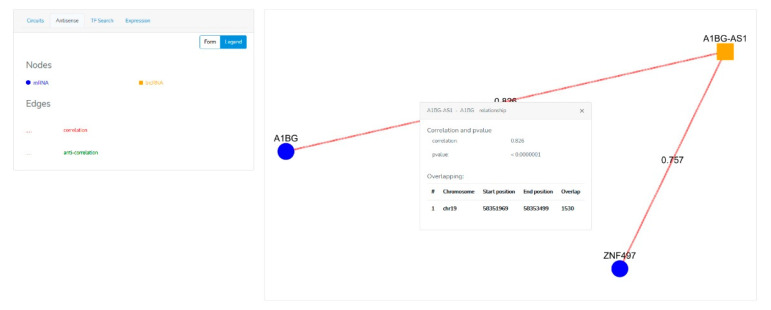
Results section of the VECTOR database for the “Antisense” query. Output data based on gene overlapping and expression correlation are depicted as an interactive network. A pop-up window appears by clicking on the edge and shows expression correlation, *p*-value and details about the overlapping of the mRNA lncRNA pair.

The Menu section (Figure 2) enables a user to provide the searching parameters through the following query types:

- The “Circuits” menu allows users to look for the molecular axes generated by lncRNA-mRNA-miRNA correlations. Users have to provide the name of at least one element that is part of the circuit (official gene symbol for mRNAs and lncRNAs, miRBase ID for miRNAs) (Figure 2A, red rectangle), and the minimum correlation coefficient of the miRNA-mRNA, lncRNA-mRNA and miRNA-lncRNA pairs. Alternatively, users can filter the output by p-value. (Figure 2A, yellow rectangle). The last parameter, named “Top n” (Figure 2A, green rectangle), limits the number of returned “triangular RNA circuits” in order to ensure a better readability of the plotted results, as well as a shorter processing time.
- The “Antisense” menu enables users to look for the sense-antisense sequence overlapping between mRNAs and lncRNAs. In this case, the user has to provide the mRNA and/or lncRNA name (Figure 2B).
- The “TF search” menu enables users to extract from our database the TF-mRNA, TF-miRNA and TF-lncRNA pairs in terms of correlation data and ChiPseq information about a given transcription factor. Therefore, the user has to provide the TF name (official gene symbol) and/or the name of a lncRNA, miRNA, and/or mRNA (Figure 2C) before submitting the search form.
- The “Expression” menu allows users to evaluate the expression levels of a selected mRNA, lncRNA or miRNA in the UM TCGA dataset and in several physiological tissues. The users have to choose the RNA molecule for which expression values in both UM and physiological tissues will be shown as histograms. To infer the potential association between the expression of a specific RNA molecule in UM and the clinicopathological parameters of UM patients, the users can select the intended parameter and VECTOR will return a new histogram graph, where UM samples are shown grouped for the selected parameter.
- The Results section (Figure 3) plots the obtained results as a network or a table.
- Once the “Circuits” or “Antisense” searching query is submitted, results will be shown through an interactive network comprising nodes and edges (Figure 3 and Figure 4). The nodes represent the RNA species: the mRNAs are shown with blue circles, the miRNAs with red triangles, and the lncRNAs with orange squares. These can be inspected (by clicking on them) to get a table listing all the TFs they interact with. The edges represent the relationships between two RNA molecules (i.e., expression correlation and potential physical interaction). Different styles and colors discriminate the kind of relationship between RNAs: red edges imply a positive expression correlation between two RNA elements, while green edges show the anti-correlation of an expression. In addition to the color, each edge is also marked with the correlation or anti-correlation numeric value, while the *p*-value is shown in a small pop-up window which appears by clicking on the edge. Moreover, in the “Circuits” section, when the expression relationship is confirmed by at least one of the databases (miRBase, miRTarBase, LncBase, miRcode, and Encori), the edge is depicted as a solid line; otherwise it is a dotted line. The database confirming the expression relationship is shown in a small pop-up window which appears by clicking on the edge.

Together with each circuit, VECTOR generates a heatmap showing the expression of each member of each circuit in the UM TCGA dataset. Expression values will be represented as a color-coded scale ranging from the minimum (green) to the median (black) to the maximum (red) expression value for each RNA molecule. The heatmaps are then shown below the network image.

**Figure 5:**
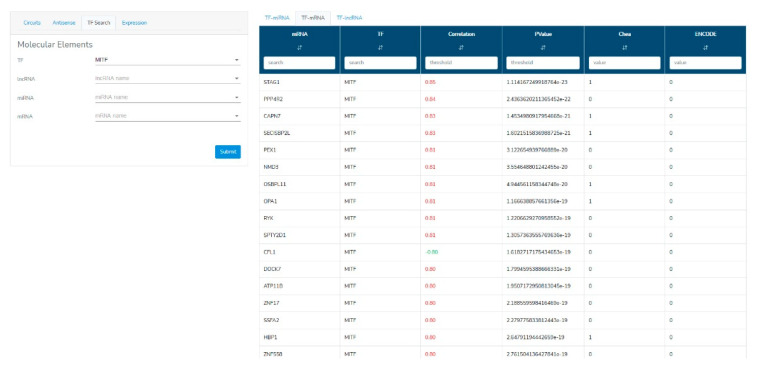
Results section of the VECTOR database for “TF search” query. The table shows expression correlation among the TF and the RNA species, with the associated *p*-value. The presence of an experimentally validated TF binding site according to Chea and ENCODE is reported as 1 (yes) or 0 (no).

**Figure 6:**
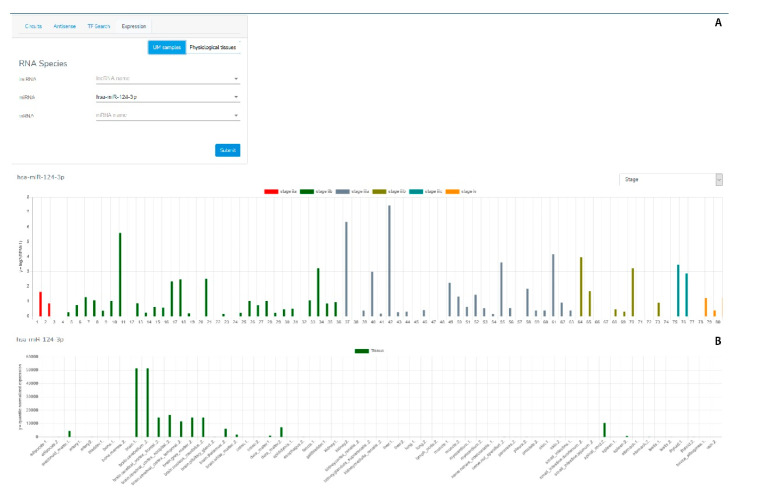
Results section of the VECTOR database for the “Expression” query. (**A**) The expression of the selected RNA molecule in UM samples from TCGA is shown as histograms. The color of the histogram allows to classify each sample according to the selected clinicopathological parameter. (**B**) The expression of the selected RNA molecule in a set of physiological tissues is shown as histograms.

- When a “TF searching” query is submitted, the obtained records are shown in a tabular format (Figure 5). Such tables contain the TF-mRNA, TF-lncRNA, or TF-miRNA expression correlations and potential physical interactions between TFs and gene promoters. TF binding to the promoter is linked to ChIPseq data from ENCODE, ChEA, and TransmiR, reported as a binary table (i.e., 0: no ChIPseq data; 1: ChIPseq data demonstrating the TF interaction on the gene promoter).
- Expression of the chosen RNA molecule in both the UM TCGA dataset and physiological tissues is shown as a histogram (Figure 6). For the UM expression data, samples are shown grouped according to a specific clinicopathological parameter, which can be selected by the user. Selecting a different parameter, samples will be reorganized in order to group all samples sharing that clinical feature. For numerical parameters, samples are shown in ascending order for the parameter. This function will allow users to observe potential expression trends for a chosen RNA molecule in association with a specific clinical feature.

## 3. Results

### 3.1. Global Identification of lncRNA–miRNA–mRNA Axes in UM

**Figure 7:**
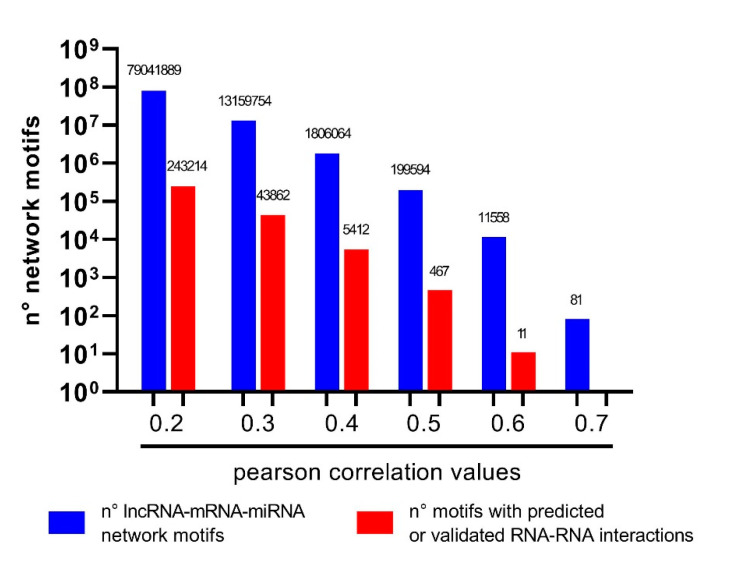
Generation of lncRNA–miRNA–mRNA axes stored in VECTOR. LncRNA–miRNA–mRNA axes (triangle-shaped network motifs) were computed by using different correlation coefficient thresholds (blue bars) and matched with the same network motifs also featuring the predicted or validated interactions in the miRNA–lncRNA and miRNA–mRNA axes (red bars).

Following our filtering approach, we built correlation matrices made up of 14,500 mRNAs, 733 lncRNAs, and 612 miRNAs. We obtained from three different matrices (i.e., mRNAs:miRNAs, mRNAs:lncRNAs, lncRNAs:miRNAs) 8,568,000, 10,628,500 and 448,596 correlation coefficients, respectively. All possible Mrna–miRNA pairs from correlation matrices were matched with mRNA–miRNA interaction data from TarBase and miRTarBase, while all lncRNA–miRNA couples were matched with lncRNA–miRNA interaction data from miRcode, lncBase and Encori. By this approach, we obtained multiple sets of triangle-shaped network motifs composed as follows: (1) miRNA–lncRNA axis (negatively correlated and interacting with each other), (2) miRNA–mRNA axis (negatively correlated and interacting with each other), (3) lncRNA–mRNA axis (positively correlated RNAs). According to the correlation thresholds applied to all pairs of the network motifs (from |0.2| to |0.7| for both positive and negative correlations), we generated different numbers of network motifs, as shown in Figure 7. By using a low-moderate threshold (correlation coefficient >0.4 or <−0.4), we obtained 1,806,064 correlation-based lncRNA-miRNA-mRNA network motifs, 5412 of which were characterized by a physical interaction according to at least one database in the miRNA–lncRNA axis and one in the miRNA–mRNA axis. The high-moderate threshold (correlation coefficient >0.5 or <−0.5) provided 199,594 correlation-based network motifs, 467 of which featuring physical interactions. The most stringent threshold (correlation coefficient >0.6 or <−0.6) featuring physical interactions between RNA molecules provided the 11 RNA network motifs reported in Table 1. The threshold correlation coefficient >0.7 resulted in 81 lncRNA–miRNA–mRNA network motifs that did not show predicted or validated RNA–RNA interactions.

### 3.2. Relationship Between Genomic Overlapping and Expression of Sense-Antisense Transcript Pairs

**Figure 8:**
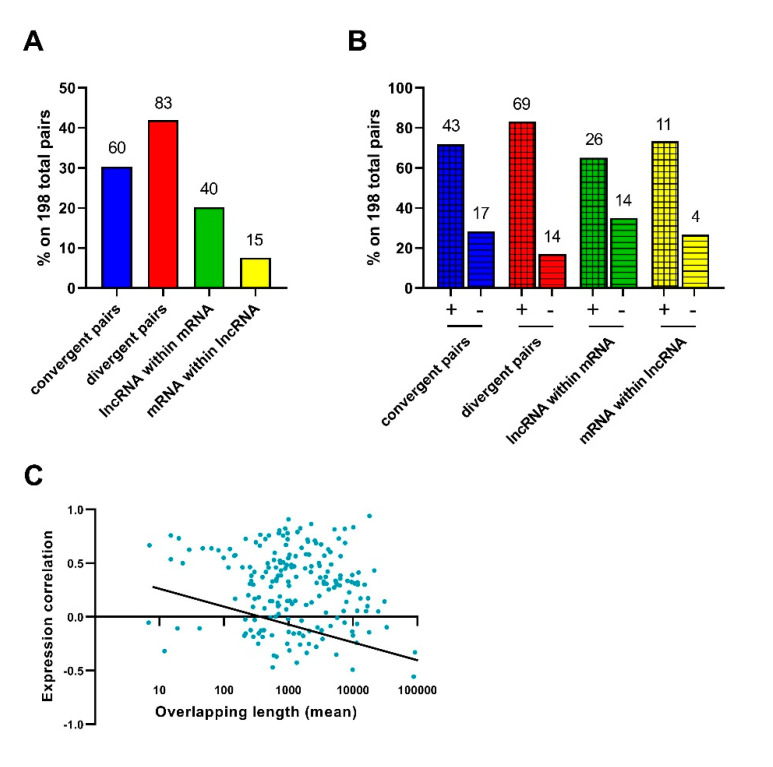
Sense-antisense lncRNA:mRNA overlapping pairs in UM. (**A**) Sense-antisense pairs were classified according to overlapping features in: convergent (lncRNA and mRNA overlap in their 3′ regions), divergent (lncRNA and mRNA overlap in their 5′ regions), lncRNA within mRNA (a shorter lncRNA totally overlapping a longer mRNA) and mRNA within lncRNA (a shorter mRNA totally overlapping a longer lncRNA). (**B**) Sense-antisense pairs divided in subgroups according to the direction of expression correlation: positive correlation is represented as a checked fill pattern of the histogram, negative correlation as a striped fill pattern. Data are shown as percentage calculated on the total number of 198 pairs; the number of pairs included in each overlapping class or subgroup is reported above each histogram. (**C**) Correlation between length of overlapping regions (mean) and expression correlation among sense and antisense transcripts.

The overlapping of genomic positions of lncRNAs and mRNAs and its combination with expression correlation data allowed us to explore the possibility of UM expression regulation mediated by sequence complementarity between genes that partially share the same locus on opposite DNA strands. Genomic positions of mRNAs were superimposed on those of lncRNAs, obtaining 198 matches. Specifically, 143 lncRNA:mRNA pairs (72.2%) showed partial overlapping, while 55 (27.8%) included one shorter locus that totally overlapped a longer one. Considering all 198 pairs, 60 pairs (30.3%) overlapped in the 3′ regions (convergent pairs), 83 (41.9%) overlapped in the 5′ regions (divergent pairs), 40 (20.2%) included lncRNA loci totally overlapped the mRNA loci, and 15 (7.6%) mRNA loci totally overlapping lncRNA loci (Figure 8A). We also divided each overlapped the class into subgroups according to the trend of expression correlation (i.e., positive and negative correlations), and observed that lncRNA and mRNA pairs mostly showed a positive rather than a negative expression correlation, particularly in divergent pairs (Figure 8B).

To investigate whether the percentage of sequence overlapping affects the direction of expression correlation between lncRNA and mRNA, we calculated the Pearson coefficient between the expression correlation values and the number of overlapping bases. For pairs including multiple splicing variants for lncRNA and/or mRNA, the length of overlapping regions was calculated as mean, minimum and maximum; in these cases, three correlation coefficients were computed. We observed a negative correlation between the expression correlation and all length values of overlapping regions: mean: −0.23; minimum: −0.19; maximum: −0.23 (Figure 8C). These results would suggest that the lower the overlapping length for two sense-antisense sequences, the stronger their expression correlation.

### 3.3. The Genome-Wide Identification of TFs Regulating mRNAs, lncRNAs and miRNAs in UM

**Figure 9:**
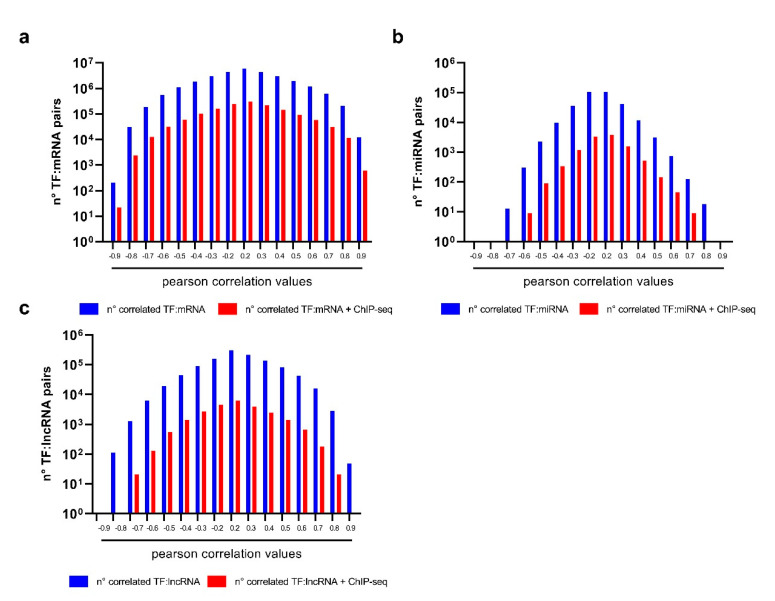
Transcription factors and their potential targets in uveal melanoma retrieved by VECTOR. TFs regulating (**a**) mRNA coding genes, (**b**) miRNA coding genes, (**c**) lncRNA coding genes are reported according to correlation coefficients as the number of correlated TF:xRNA pairs (blue bar) and the number of correlated TF–xRNA pairs whose TF potentially binds the promoter of xRNA genes (red bar).

We built three different correlation matrices made up of (1) 1278 TFs and 14,500 mRNAs; (2) 1278 TFs and 612 miRNAs; and (3) 1278 TFs and 733 lncRNAs. By this approach, we obtained 1,853,1000 correlation coefficients for the TF:mRNA matrix, 782,136 for TF–miRNA matrix, and 936,774 for TF–lncRNA. All TF–xRNA couples were screened for binding of TFs on promoters of xRNA genes (xRNA = any type of RNA), according to ChiPseq data from ENCODE, ChEA, and TransmiR. Finally, we obtained several sets of positively or negatively correlated TF–xRNA pairs, whose TF potentially binds the promoter of xRNA genes (Figure 9). According to the most stringent negative and positive correlation coefficient thresholds and the presence of ChiPseq hits, we retrieved potential UM TF–target pairs. More specifically, we obtained 631 TF–mRNA, 18 TF–miRNA, 42 TF–lncRNA pairs. From these data, we extrapolated the 23 most frequent TFs regulating mRNAs, miRNAs and lncRNAs in UM (Table 2). Unsurprisingly, most TFs reported in Table 2 have a confirmed oncogenic role in different types of neoplasia, including melanoma.

The most frequent TFs were retrieved by using the most stringent and evaluable parameters of TF querying by VECTOR (TF–mRNA = correlation coefficient <−0.9 and >0.9; TF–miRNA = correlation coefficient <−0.6 and >0.7; TF–lncRNA = correlation coefficient <−0.7 and >0.8). From this output, the TFs shows that at least three targets were retrieved. The negative expression correlation between TFs and targets are indicated with a minus symbol between brackets (-) next to the target name. / = no data. Literature regulation mechanisms are highlighted in bold; references reporting the role in cancer or the regulation of the transcript are shown as PubMed IDs.

## 4. Discussion

In the last decade, a growing number of experimental studies have demonstrated that RNA-RNA crosstalk is implicated in cell-fate determination and in various human diseases, including cancer. CeRNA mechanisms are able to modulate concentration and functions of RNA elements from specific molecular axes and, accordingly, regulate essential biological processes [9,30]. The combination of expression relationships among RNA molecules and their complementarity-based binding provides a reliable scenario of RNA network structure and represents a pivotal starting point for planning experimental procedures to validate and functionally analyze RNA circuits. This methodological approach is very common in most studies concerning the role of lncRNAs in cancer: researchers retrieve expression and interaction data from different public databases and then compute and integrate them to obtain an RNA signaling to experimentally evaluate [31,32,33,34]. This computational method could take a few hours, depending on the amount of information available for a specific cancer model and the researchers’ expertise. Based on these considerations, we created VECTOR, a simple and intuitive database containing the elaboration and integration of expression correlations and experimental and predicted interactions among lncRNAs, miRNAs and mRNAs. Users are allowed to inspect expression of mRNAs, lncRNAs or miRNAs not only in the UM TCGA dataset, but also in other physiological tissues, to evaluate ubiquitarity or specificity of expression. Expression data are shown as histograms. Additionally, VECTOR allows to observe potential association trends between expression and clinical features of UM patients: indeed, the expression data of UM samples can be shown by grouping or ordering samples according to a selected clinicopathological parameter. This option allows to observe an increasing/decreasing expression trend in association with tumor stage, tumor size (thickness or basal diameter), metastasis, and other features. To investigate ceRNA networks, the query can be customized by choosing the type and magnitude of correlations. The relationships among RNAs are visualized as graphs featuring information about correlation and physical interaction in order to make the output easier to understand; expression of each member of the network are represented below as heatmaps. In addition to the classical ceRNA hypothesis view based on the lncRNA-miRNA-mRNA axis, VECTOR also queries the relationships between sense-antisense transcripts and the potential transcriptional regulations by transcription factors in order to obtain a more systemic view of RNA signaling. The choice of UM as the cancer model of VECTOR was dictated by the fact that it is a rare tumor and that there are not many studies on the ncRNAs involved in the onset and progression of this neoplasm. Thanks to UM TCGA findings, it is now possible to statistically elaborate these data to identify the expression relationships among RNA molecules and correlative interaction data from other sources. Other previous studies reported a systemic integrated view of RNA correlations in UM [35,36]; however, in this present work we propose the most comprehensive analysis and make these elaborations available through an interactive web-based user interface. The approach used to build VECTOR was already used in our previous papers on both UM and other cancer models [12,15,37]. In these previous studies, data about expression, interactions, and potential regulation operated by TFs were retrieved from different sources and databases, making this analysis complicated and time-consuming. The great advantage of VECTOR is the possibility to perform this analysis with only a few clicks. The strength of our approach is given by several matches observed between results obtained by using VECTOR and literature evidence. Indeed, among the triangle-shaped network motifs resulting from the application of the most stringent threshold of expression correlation, a molecular axis involving a miRNA sponge function of LINC00518 (long intergenic non-protein coding RNA 518) for miR-199a-5p was already reported in breast cancer [38]. Moreover, our previous paper on UM also suggested that LINC00518 may exert a miRNA sponge function on six miRNAs, including miR-199a-5p [15]. Similarly, several TFs identified by VECTOR as potential regulators of mRNA expression are confirmed by reports in the literature: an autoregulative loop was previously reported for CREB1 (cAMP responsive element binding protein 1) [39,40], GABPA (GA binding protein transcription factor subunit α) [41,42,43], JUND (JunD proto-oncogene, AP-1 transcription factor subunit) [44], MAZ (MYC associated zinc finger protein) [45], RELA (RELA proto-oncogene, NF-kB subunit) [46], SOX2 (SRY-box transcription factor 2) [47,48], and SPI1 (Spi-1 proto-oncogene) [49,50,51]. Additionally, CREB1 was shown to induce MEF2A (myocyte enhancer factor 2A) expression in human trophoblast stem cells and T-cells [52,53].

Another feature of VECTOR is the possibility to identify sense-antisense pairs to experimentally investigate their expression relationship. This aspect has been investigated since the early 2000s, and several papers have reported that the majority of sense-antisense pairs exhibit a positive correlation of expression [54,55,56,57,58]. A recent study showed that a positive correlation occurred in divergent pairs more frequently than in convergent pairs, likely because head-to-head overlap implies that genetic loci share a region with an open chromatin structure and the same regulation; on the contrary, convergent pairs showed both a positive and negative correlation [59]. Our analysis confirmed these observations in UM, where a positive correlation of expression is the most common in all the overlapping classes, but especially in divergent pairs (more than 80% showing a positive correlation of expression). Moreover, in agreement with the literature, a negative correlation was more frequent among convergent than divergent pairs (28.3% vs. 16.8%, respectively). The exact molecular mechanism underlying the co-regulation or inverse correlation of sense and antisense transcripts is still under investigation. Some studies showed that transient silencing of sense or antisense transcript did not affect the expression of the other one [60], while other papers demonstrated that the antisense transcript is responsible for the regulation of sense transcript expression at both RNA [61] and protein levels [62]. This evidence suggests very complex regulatory mechanisms that still need to be investigated. We also analyzed expression correlation for pairs including a shorter RNA molecule that totally overlapped a longer transcript. Our data showed that lncRNAs totally overlapping mRNAs are more frequent that mRNAs totally overlapping lncRNAs (40 vs. 15, respectively), with a slightly higher frequency of negative correlation in the first class (35% vs. 26.7%, respectively). For these two overlapping classes, we investigated whether overlapping regions fell within introns or exons, but the existence of multiple splicing variants for both sense and antisense transcripts created a very complex and heterogeneous scenario, where no clear classification (and consequent analysis) was possible. To our knowledge, no similar analysis has been performed to date on human cancer.

VECTOR was built according to our experience in ceRNA network investigations. However, some limits deriving from the available data should be discussed. The most evident limit is related to the type of samples included in the UM TCGA dataset. Indeed, when investigating the molecular bases of carcinogenesis, a researcher would first of all perform a comparison between tumor and normal tissue to identify differentially expressed molecules. Unfortunately, the UM TCGA dataset includes tumor tissue samples, but lacks in data on physiological tissue. Therefore, such an analysis is impossible to perform with this dataset. Other interesting analyses could be performed through the stratification of tumor samples according to clinicopathological data; however, stratification would create several subgroups consisting of a low number of samples, impairing the statistical power of such analysis. For this reason, stratified analyses were not performed, but VECTOR allows the users to inspect expression trends in the different subgroups obtained by stratifying samples for the available clinicopathological features of patients.

## 5. Conclusions

Comprehensively, VECTOR provides a very detailed picture of ceRNA networks in UM and we believe that it will represent a very useful tool for researchers studying RNA signaling in UM. Moreover, the VECTOR approach could be used to build other tools for different cancer models in order to make the tumor-related ceRNA circuits easily accessible to non-expert biocomputational researchers willing to devise an experimental plan.

## Figures and Tables

**Table 1:** LncRNA–miRNA–mRNA axes featuring physical interactions calculated by using the most stringent threshold (correlation coefficient > 0.6).

miRNA	Pearson miRNA–mRNA	mRNA	TarB	mirTar	lncRNA	Pearson miRNA–lncRNA	miRc	lncB-V	lncB-P	En	Pearson lncRNA–mRNA
hsa-miR-199a-5p	−0.65	CDCA7L	1	0	LINC00518	−0.66	1	0	0	0	0.65
hsa-miR-199a-5p	−0.65	CDCA7L	1	0	SNHG7	−0.69	1	0	0	0	0.73
hsa-miR-195-5p	−0.60	SDC3	1	0	LINC01128	−0.67	0	0	0	1	0.72
hsa-miR-199a-5p	−0.66	RPL15	1	0	LINC00518	−0.66	1	0	0	0	0.64
hsa-miR-199a-5p	−0.66	RPL15	1	0	SNHG7	−0.69	1	0	0	0	0.80
hsa-miR-199a-5p	−0.66	RPL15	1	0	WDFY3-AS2	−0.63	1	0	0	0	0.62
hsa-miR-199a-5p	−0.63	ZNF415	0	1	LINC00518	−0.66	1	0	0	0	0.72
hsa-miR-195-5p	−0.66	TPRG1L	1	0	LINC01128	−0.66	0	0	0	1	0.82
hsa-miR-508-3p	−0.61	GPR176	0	1	HCP5	−0.70	1	0	0	0	0.63
hsa-miR-195-5p	–0.65	BSDC1	1	0	LINC01128	–0.67	0	0	0	1	0.61
hsa-miR-195-5p	–0.65	CTNNBIP1	1	0	LINC01128	–0.67	0	0	0	1	0.72

lncRNA-miRNA-mRNA network motifs were calculated by (1) retrieving the miRNA–mRNA, miRNA–lncRNA, and lncRNA–mRNA pairs with correlation coefficients of <−0.6 and >0.6, respectively; (2) identifying miRNA–mRNA and miRNA–lncRNA axes with at least one predicted or validated interaction from Tarbase (TarB), miRTarBase (mirTar), miRcode (miRc), lncBase (lncB-V: validated modules; lncB-P: predicted modules), and Encori (En).

**Table 2:** The most frequent TFs regulating mRNA, miRNA and lncRNA coding genes in UM according to the most stringent parameters of VECTOR.

TFs	mRNAs	miRNAs	lncRNAs	Role in Cancer
CDC73	ATF2, BACH1, CREB1, ELF1, MEF2A	/	/	Oncogene (29221126)/tumor-suppressor (24145611)
COG6	BACH1, CREB1, ELF1, GABPA	/	/	/
CREB1	CREB1 (15340044, 9790528), MEF2A (26606046, 25809782)	/	SEPT7P2, SUZ12P1, ZNF252P, ZNF37BP	Oncogene (17786359, 28498439, 27801665)
EPC2	ATF1, BACH1, CREB1, ELF1, MEF2A, SMAD4	/	/	Oncogene (24166297)
GABPA	GABPA (17277770, 21139080, 16309857)	/	LOC407835(-), CCT6P1, LOC100190986, SUZ12P1, ZNF37BP	Tumor-suppressor (31802036, 28549418)
JUND	JUND (8172655)	/	FAM35BP(-), FAM35DP(-)	Oncogene (30763715, 27358408)/tumor-suppressor (18454173)
MAZ	MAZ (11259406)	/	BDNF-AS(-), CCT6P1(-), SBDSP1(-), SEPT7P2(-)	Oncogene (31488180, 29414775)
MORC3	BACH1, CREB1, ELF1, GABPA	/	/	/
NARFL	ATF2(-), BACH1(-)	/	/	/
PIKFYVE	ATF2, CREB1, MEF2A, ZFX	/	/	Oncogene (17909029, 24840251, 23154468)
RELA	RELA (24425788)	/	SEPT7P2(-), SNHG10(-), ZNF37BP(-)	Oncogene (17622249, 12615723)/tumor-suppressor (11747334)
SF3B1	ATF2, BACH1, CREB1, ZNF143	/	/	/
SOX2	SOX2 (16153702, 12136102)	hsa-miR-124-3p, hsa-miR-183-5p, hsa-miR-96-5p	/	Oncogene (31412296, 31748974, 30518951)
SP3	ATF2, CLOCK, CREB1, MEF2A, YY1	/	/	Oncogene (20810260, 26967243, 26352013)
SPI1	SPI1 (7478579, 15767686, 20190819)	hsa-miR-146b-3p, hsa-miR-146b-5p, hsa-miR-150-5p	LOC606724, NCF1B, NCF1C	Oncogene (28415748)
TFAP2A	/	hsa-miR-145-3p(-), hsa-miR-199a-5p(-), hsa-miR-4709-3p(-), hsa-miR-708-5p(-), hsa-miR-887-3p(-), hsa-miR-937-3p(-), hsa-miR-181a-5p	/	Oncogene (31772149, 30824562, 28412966)/tumor-suppressor (30824562)
TRAPPC8	ATF1, ATF2, CREB1, MEF2A, YY1	/	/	/
USF2	USF2	/	SBDSP1(-), SEPT7P2(-)	Oncogene (30244169)/tumor-suppressor (16186802)
XPO1	ATF2, BACH1, CEBPZ, CREB1	/	/	Oncogene (32487143, 30976603, 24431073)
ZBTB45	CREB1(-), MEF2A(-)	/	/	
ZFR	ATF2, BACH1, CLOCK, CREB1, ELF1, YY1	/	/	Oncogene (31010678)
ZNF143	ZNF143	/	LOC407835(-), SEPT7P2, SUZ12P1, ZNF37BP	Oncogene (27449034, 20860770, 32312832)
ZNF791	ATF2, BACH1, CREB1, SP4, YY1	/	/	/

The most frequent TFs were retrieved by using the most stringent and evaluable parameters of TF querying by VECTOR (TF–mRNA= correlation coefficient <−0.9 and >0.9; TF–miRNA = correlation coefficient <−0.6 and >0.7; TF–lncRNA= correlation coefficient <−0.7 and >0.8). From this output, the TFs shows that at least three targets were retrieved. The negative expression correlation between TFs and targets are indicated with a minus symbol between brackets (-) next to the target name. / = no data. Literature regulation mechanisms are highlighted in bold; references reporting the role in cancer or the regulation of the transcript are shown as PubMed IDs.

## Data Availability

The web version of Vector is freely available at the following URL: https://vectordb.it/.
